# Influence of thermal denaturation on whey protein isolates in combination with chitosan for fabricating Pickering emulsions: a comparison study

**DOI:** 10.3389/fnut.2024.1418120

**Published:** 2024-06-03

**Authors:** Yilin Pu, Yuxiang Long, Die Xu, Yongkang Niu, Qinglong Wu, Shiyu Chen, Ruozhen Wang, Ruihong Ge

**Affiliations:** ^1^College of Basic Medical Sciences, Shanghai Jiaotong University School of Medicine, Shanghai, China; ^2^School of Public Health, Shanghai Jiao Tong University School of Medicine, Shanghai, China

**Keywords:** thermally denatured whey protein isolate, Pickering emulsions, interfacial tension, rheological properties, stability mechanism

## Abstract

Composite natural emulsifiers such as whey protein isolate (WPI) and chitosan (CS) are commonly used in Pickering emulsions to address the effect of thermal deformation of proteins before complexation with CS and heating after complexation. In this study, the properties of WPI and CS composites were investigated by complexing CS with either unmodified WPI or thermally denatured WPI (DWPI). Three types of composite particles were prepared, WPI-CS, DWPI-CS, and D(WPI-CS). Atomic force microscopy revealed that the composite particles formed larger aggregates with increased contour size and surface roughness compared to CS and WPI, whereas the interfacial tension decreased, indicating improved emulsifying abilities. Fourier-transform infrared analysis revealed differences in the hydrogen bonds between CS and WPI/DWPI. All three composite particles formed stable emulsions with droplet sizes of 20.00 ± 0.15, 27.80 ± 0.35, and 16.77 ± 0.51 μm, respectively. Thermal stability experiments revealed that the curcumin emulsion stabilized with WPI-CS and DWPI-CS exhibited relatively better thermal stability than that stabilized with D(WPI-CS). *In vitro* experiments results indicated that the bioaccessibility of the curcumin emulsion stabilized with WPI-CS was 61.18 ± 0.16%, significantly higher than that of the emulsions prepared with the other two composite particles (*p* < 0.05). This study will enable the customized design of WPI composite-based Pickering emulsions for application in the food and nutrition industries.

## 1 Introduction

Pickering emulsions are emulsions in which nanometer-sized solid particles stabilize immiscible fluids. Owing to their advantages, such as low toxicity, good anti-coalescence, and potential applications in the delivery of bioactive substances, they have attracted significant attention in food, medicine, and other fields (1, 2). Numerous natural ingredients such as protein-based materials (whey protein, soy protein, and gelatin), polysaccharide-based materials (chitin, starch, and cellulose), and their composites have been used as substitutes for synthetic surfactants (3).

Whey protein isolate (WPI) contains 90–96% functional protein (4), mainly consisting of β-lactoglobulin (60–70%), a small amount of α-lactalbumin, and bovine serum albumin (5). WPI is widely used in the food industry because of its high nutritional value and excellent functional properties, such as emulsification, foaming, and gelling behaviors (6–8). Owing to its natural, extensive sources and GRAS (generally recognized as safe) status (8), it has been used extensively in multiple drug applications and biological systems (9, 10). WPI can be combined with a variety of nutritional drugs with different affinities and specificities through hydrogen bonding, hydrophobic interactions, and van der Waals forces, and it is efficient as a nanocarrier or a key component in the construction of nanocarriers to stabilize and deliver functional components (8).

Chitosan (CS) is a natural linear cationic polysaccharide usually obtained through the alkaline or enzymatic treatment of deacetylated chitin. Owing to its promising properties such as excellent biodegradability and biocompatibility, ready availability, low cost, and nontoxicity, as well as antibacterial, antifungal, antitumor, and antihypertensive activities, it has attracted increasing attention in the food industry (11). Additionally, it has attracted significant attention because of its novel role in stabilizing O/W emulsions and preparing nanocarriers and microgels by constructing polyelectrolyte complexes with negatively charged macromolecules.

Previous studies have reported that emulsions with a single emulsifier are unstable (9, 10). For example, emulsions coated with CS alone are unstable (12), and oil droplets stabilized by WPI are particularly sensitive to pH levels, aqueous-phase ionic strength, and heat treatment (6). The stability of WPI-stabilized oil emulsions is affected by processing and storage conditions during use (13, 14). However, proteins and polysaccharides are natural biopolymers with abundant sources. The assembly of protein and polysaccharide complexes may help establish an economically feasible delivery system with high biocompatibility and biosafety (9, 10). Composite emulsifiers can increase the electrostatic or spatial repulsion between oil droplets by enabling the formation of interfacial complexes, thereby improving the stability of oil droplets. Compared to proteins or polysaccharides alone, WPIs and CS complexes have synergistic stabilizing effects on emulsions. In addition to electrostatic or spatial repulsions, hydrogen bonding, van der Waals interactions, and hydrophobic phase interactions occur between proteins and polysaccharide complexes. Apart from providing greater stability for the emulsion interface, a delivery carrier based on proteins and polysaccharides can also protect the bioactive components in the inner phase of the emulsion from environmental stresses (such as light and oxygen). Therefore, protein and polysaccharide complexes are widely used to prepare emulsion delivery systems (15).

WPI is often modified in the food industry to enhance its functional properties (16) with heat treatment being the most common modification method. Preheating a WPI dispersion directly affects the stability of the final emulsion (17). When heated to a temperature higher than the denaturation temperature, the protein molecules of the WPI solution or β-lactoglobulin suspension can expand and expose their hydrophobic and thiol groups, forming irreversible protein aggregates (18, 19). When heated at a neutral pH, the molecular structures of WPI, especially β-lactoglobulin and α-lactalbumin, can alter their physical and chemical reactions (such as electrostatic and hydrophobic interactions and disulfide bonding), increasing the viscosity and surface hydrophobicity of the WPI dispersions (20). Under these conditions, the particle size of the final emulsion decreases and the stability increases (17).

Previous studies have shown that the controlled heating or heat setting of whey protein and polysaccharide complexes produces submicron particles and stabilizes them to resist pH changes (21). This process depends on the thermal denaturation of proteins and their electrostatic interactions with polysaccharides (22). This phenomenon may occur due to the structural recombination caused by heat treatment; this alters the deposition of amino acid residues (23) or polysaccharides exposed to solvents outside the denatured protein aggregates (24), which favors the existence of a higher polysaccharide charge on the particle shell (23, 25). Consequently, the higher surface charge increases the electrostatic stability and prevents particle aggregation (26, 27).

Kotchabhakdi and Vardhanabhuti (22) revealed that unheated WPI-pectin complexes (CPXs) and heated WPI-pectin complexes (HCPXs) could be successfully adsorbed at the oil-in-water interface and improve its emulsification properties, with HCPXs exhibiting a higher negative charge and smaller droplet size. All emulsions prepared by the two composites were stable; however, the one formed by HCPXs at 85°C was more heat-stable (22). Numerous studies have focused on Pickering emulsions stabilized by WPI-CS complexes, whereas others have used denatured whey protein isolate (DWPI) and CS (28), or a combination of undenatured WPIs and CS (29–31). These studies focused on evaluating the properties of the emulsion stabilized by the composite particles and few have compared the composite particles prepared in the aforementioned ways and the resulting Pickering emulsions, including the differences in the stability of the Pickering emulsions depending on the different composites. In addition, the effect of thermal deformation of proteins before complexation and heating after complexation, the interaction between particles, and the mechanism of their action are yet to be elucidated.

This study aimed to elucidate the interactions between CS and WPIs induced by heat treatment and reveal the differences in the formation and stability mechanism of emulsions prepared by CS, WPI, and DWPI complexes before and after heat treatment. To this end, WPI-CS and DWPI-CS particles were prepared by complexing WPI or DWPI with CS, respectively. D(WPI-CS) was prepared by complexing WPI with CS, followed by heat treatment. The particle size, ζ potential, Fourier-transform infrared (FTIR) spectra, X-ray diffraction (XRD) patterns, and other parameters of the three composite particles were analyzed, and their microstructures were characterized using scanning electron microscopy (SEM) and atomic force microscopy (AFM). The interfacial tension between the composite particles and oil was measured to reveal the effect of heating on the emulsification properties of these particles. Pickering emulsions were constructed using these particles as the water phase and medium-chain triglycerides (MCT) as the oil phase. The prepared Pickering emulsions were evaluated for their particle size, ζ potential, microstructure, and rheological properties. Our study provides insights into the interaction between WPI and CS before and after heating and the ways these components affect the functional properties and stability of the protein. Our results offer a valuable tool for designing customized WPI composite-based Pickering emulsions for application in the food and nutrition industries.

## 2 Materials and methods

### 2.1 Materials

CS, with a deacetylation value of >80.0% and a viscosity of 5–20 mPa s, was provided by Tokyo Chemical Industry Co., Ltd. WPI and MCT were provided by Shanghai Yuanye Biotechnology Co., Ltd.; the protein content of WPI was 85%. Other reagents (analytical purity) were purchased from China National Pharmaceutical Chemical Reagent Co., Ltd.

### 2.2 Preparation of CS stock solution

CS (7.5 g) was dissolved in 500 mL of a 1.0% (v/v) acetic acid solution and stirred for 4 h using a stirrer (IKA Eurostar 40 Digital Mixer, IKA, Germany) at 800 rpm to prepare a 1.5% (w/v) CS stock solution. The solution was maintained at 4°C for 12 h before use for complete dissolution and hydration (32).

### 2.3 Preparation of WPI and DWPI stock solutions

WPI (50 g) was dissolved in 500 mL of pure water and stirred evenly at 800 rpm for 30 min, followed by filtration through a 70-μm nylon filter membrane to obtain a 10% (w/v) WPI solution. Half of the WPI solution was transferred into a reagent bottle and placed in a water bath (SWT-100; MIU Lab, China) at 90°C for 30 min to obtain DWPI. The prepared WPI and DWPI solutions were stored in a refrigerator at 4°C.

### 2.4 Preparation of WPI and CS composite particles

The pH of the CS solution was adjusted to 6.8–7.0 using a 1.0 mol/L NaOH solution. Equal volumes of 1.5% CS and 10% WPI solutions were mixed and stirred continuously at 800 rpm for 45 min to obtain a composite solution with a final concentration of 0.75% CS and 5% WPI (33) (WPI-CS composite solution). Half of the WPI-CS composite solution was placed in a water bath at 90°C for 30 min to obtain the D(WPI-CS) solution. The DWPI-CS composite solution was prepared using the same WPI-CS composite method by substituting the WPI solution with DWPI.

**Figure 1:**
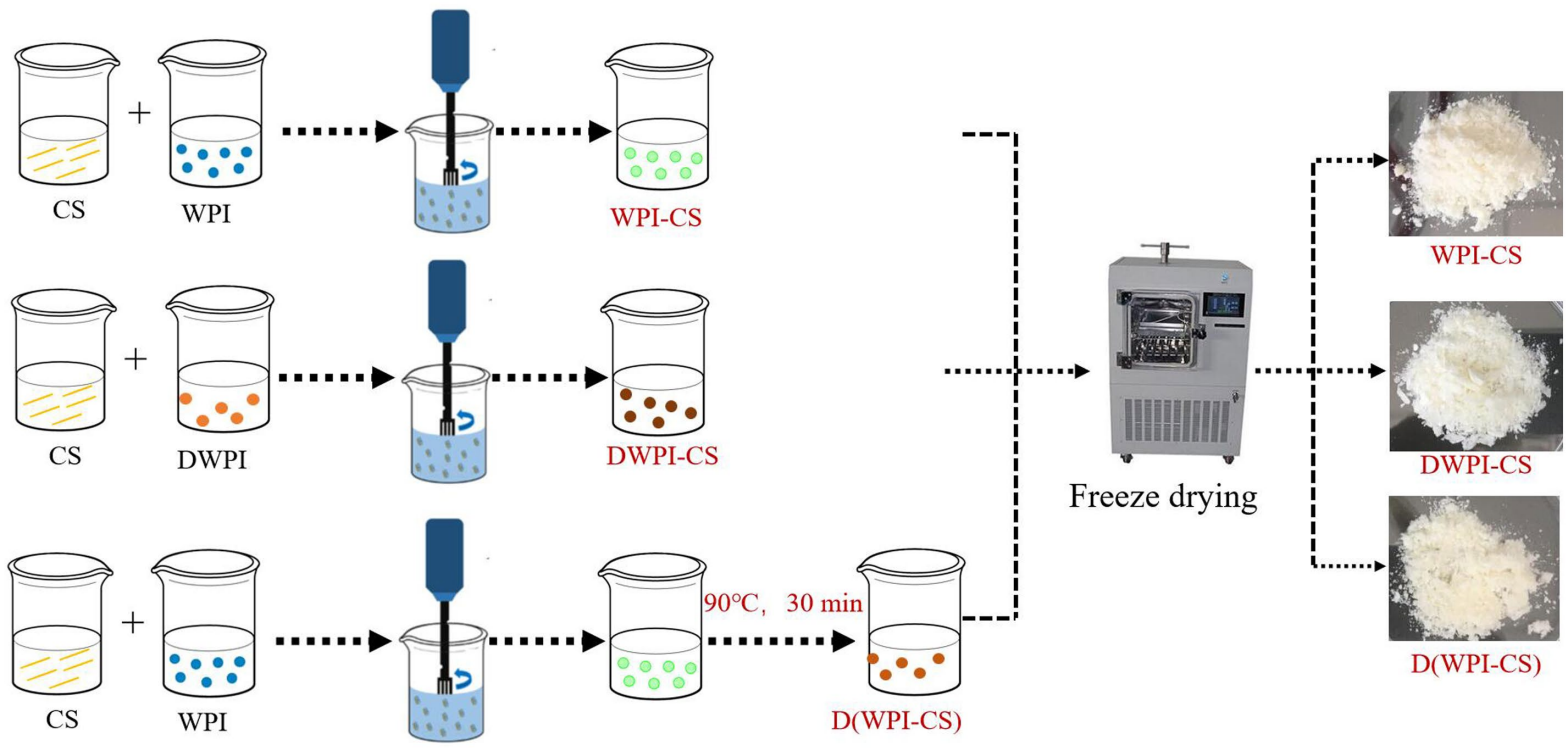
Schematic of the preparation procedure of WPI-CS, DWPI-CS, and D(WPI-CS) composite particles. The images on the right are representative SEM images of the freeze-dried particles.

The particle size, ζ potential, surface topography, and interfacial tension of the prepared composite particle solutions were analyzed. An aliquot of each solution was dried in a vacuum freeze dryer (SCIENTZ-10ND, Ningbo, Scientz Biotechnology Co., Ltd., China) for 48 h to obtain composite particle powder samples for SEM, XRD, and FTIR analyses. The preparation process for the analyses of the WPI-CS, DWPI-CS, and D(WPI-CS) composite particle samples is illustrated in Figure 1.

### 2.5 Determination of particle size and ζ potential

The particle sizes of CS, WPI, DWPI, and the three types of WPI and CS composite particle samples (WPI-CS, DWPI-CS, and D(WPI-CS)) were measured using a Malvern particle size analyzer (ZS90 nanometer size analyzer NanoZS90; Malvern Instruments Ltd., United Kingdom). The particle size of the emulsion was measured using a laser particle size analyzer (Hydro 2000SM (A), Malvern Instruments Ltd., United Kingdom). The ζ potentials of the composite particles and emulsions were determined using a Malvern particle size analyzer. Before measurement, the particle and emulsion samples were diluted in a 5 mM phosphate buffer solution (pH ~7.2). The analyses were conducted in triplicate and the average was calculated (32).

### 2.6 SEM analysis of the different WPI and CS composite particles

The morphologies of CS, WPI, DWPI, and the three types of WPI and CS composite particle samples were analyzed using SEM (Hitachi, Regulus 8100, Japan) at an accelerating voltage of 1.0 kV. Before measurement, 0.1 g of each sample was sprayed with gold for 20 s to make it conductive.

### 2.7 AFM analysis of the different WPI and CS composite particles

CS, WPI, DWPI, and the three types of WPI composite particle solution samples used for AFM analysis were prepared as described in section 2.4. These samples were diluted to 2 mg/mL in water. The diluted sample (100 μL) was placed on the surface of the mica sheet, naturally dried, and scanned using a Nanoscope V Multimode 8 scanning probe microscope (Bruker Corporation, United States). NanoScope software was used to analyze the data and draw graphs (33).

### 2.8 Measurement of interfacial tension of the different WPI and CS composite particles

The interfacial tension of the different particle types and MCT was measured using an interfacial tensiometer (K100, Kruss, Germany). A platinum ring was firstly placed into the aqueous phase (the particle solution prepared as described in section 2.4), followed by the addition of the oil phase (MCT), and the platinum ring was adjusted until the top of the ring on the two-phase interface. The surface tension was recorded at 60,000 ms and a detection speed of 6 mm/min. The system was equilibrated at room temperature for 20 min before each measurement (34).

### 2.9 XRD analysis of the different WPI and CS composite particles

The different types of particles (20 mg) were placed onto a D8 diffractometer (Bruker, Germany) for XRD detection. The scanning angle range was 5–90° and the scanning speed was 4°/min (35).

### 2.10 FTIR analysis of the different WPI-CS composite particles

The different types of particles were mixed with KBr, compressed, and subjected to FTIR analysis (Spotlight 400, PerkinElmer, Massachusetts, United States) at a wavelength range of 400–4,000 cm^−1^ (36).

### 2.11 Preparation of Pickering emulsions based on different particles

MCT was selected as the oil phase and CS, WPI, DWPI, and their composite particle solutions were added as the aqueous phase. Each emulsion was prepared by adding 8.4 mL of the oil phase and 3.6 mL of the aqueous-phase solution in a 20 mL transparent glass bottle, followed by emulsification at 14000 rpm for 3 min (Ultra Turax T18, IKA, Germany).

### 2.12 Microstructure observation of emulsions with different particles

The emulsion (5 μL) was transferred with a pipette onto a microscope slide and the sample slide was placed on a microscope (BX 53, OLYMPUS, Japan) equipped with a camera (DP74, OLYMPUS). The microstructure of the emulsion samples was observed at 20× magnification under white light.

### 2.13 Rheological properties of the emulsions with different WPI-CS composite particles

The rheological properties of the emulsion samples, including their apparent viscosity (*η*), storage modulus (*G*′), and loss modulus (*G*″), were analyzed using a rheometer (MCR302, Anton Paar, Austria). The detection method was based on a published study (37).

### 2.14 Preparation of the curcumin emulsion based on WPI and CS composite particles

Curcumin was dissolved in MCT at a concentration of 8 mg/mL (w/v). After vortex mixing, the mixture was ultrasonicated twice in a water bath (30 s each time) and then filtered through a 70 μm filter membrane to obtain the curcumin solution as the oil phase. The encapsulated curcumin emulsion stabilized with WPI and CS particles was prepared according to the method described in section 2.11.

### 2.15 Preparation of the curcumin emulsion based on Tween 80 and Span 80

An aqueous phase containing 1% Tween 80 and 1% Span 80 was mixed with the curcumin MCT solution at a ratio of 1:1 (v/v). The conventional emulsion was prepared using an IKA Ultra Turrax T18 homogenizer at 14,000 rpm for 3 min, which was used as the control group.

### 2.16 Thermal stability of the curcumin emulsion

Each group of Pickering emulsion-encapsulated curcumin was prepared in a 20 mL glass bottle and placed in a water bath shaker at 80°C. Portions of each emulsion (10 μL) were sampled using a pipette at 0, 1, 2, 3, and 4 h, and sensory evaluation and microstructure observation followed.

### 2.17 *In vitro* simulated digestion test of the curcumin emulsion

The *in vitro* gastrointestinal tract (GIT) model of this experiment included three stages: simulation of the oral cavity, the stomach, and the intestine. The simulated digestive solution was prepared according to the method described by Mulet et al. (38, 39). The *in vitro* experiments were conducted following our previously reported methods (32).

### 2.18 Bioavailability of the curcumin emulsion

The *in vitro* bioavailability experiments were conducted according to previously reported methods with some modifications (40). Following digestion in the small intestine, the digested reaction solution was ultracentrifuged at 40,000 rpm and 4°C for 60 min (Optima XPN-100, Beckman, United States) (186,000 g), and the intermediate aqueous phase containing the curcumin micelles was collected and its volume was recorded. The curcumin concentration in the micelles was analyzed following our previously reported method (32). The bioavailability percentage of curcumin was calculated using Eq. 1.

(1) $$\mathrm{Bioaccessibility}\;\left(\%\right)=\frac{\begin{array}{l} \mathrm{Amount}\;\mathrm{of}\;\mathrm{solubilized}\;\\ \mathrm{curcumin}\;\mathrm{micelle} \end{array}}{\begin{array}{l} \mathrm{Amount}\;\mathrm{of}\;\mathrm{curcumin}\;\mathrm{in}\;\\ \mathrm{original}\;\mathrm{emulsion} \end{array}}\times 100$$

### 2.19 Data analysis

The particle size distribution, interfacial tension, XRD, FTIR, and rheological results were processed and plotted using the Origin Pro software 2021. The average particle size and ζ potential results were processed using the GraphPad Prism 7.00 and MS Excel 2019 software, respectively. The results for the particle sizes, polymer dispersity index (PDI), and ζ potentials were expressed in terms of the mean ± standard deviation (Mean ± SD).

## 3 Results and discussion

### 3.1 Characterization of the composite particles

#### 3.1.1 Particle size and ζ potential

**Figure 2:**
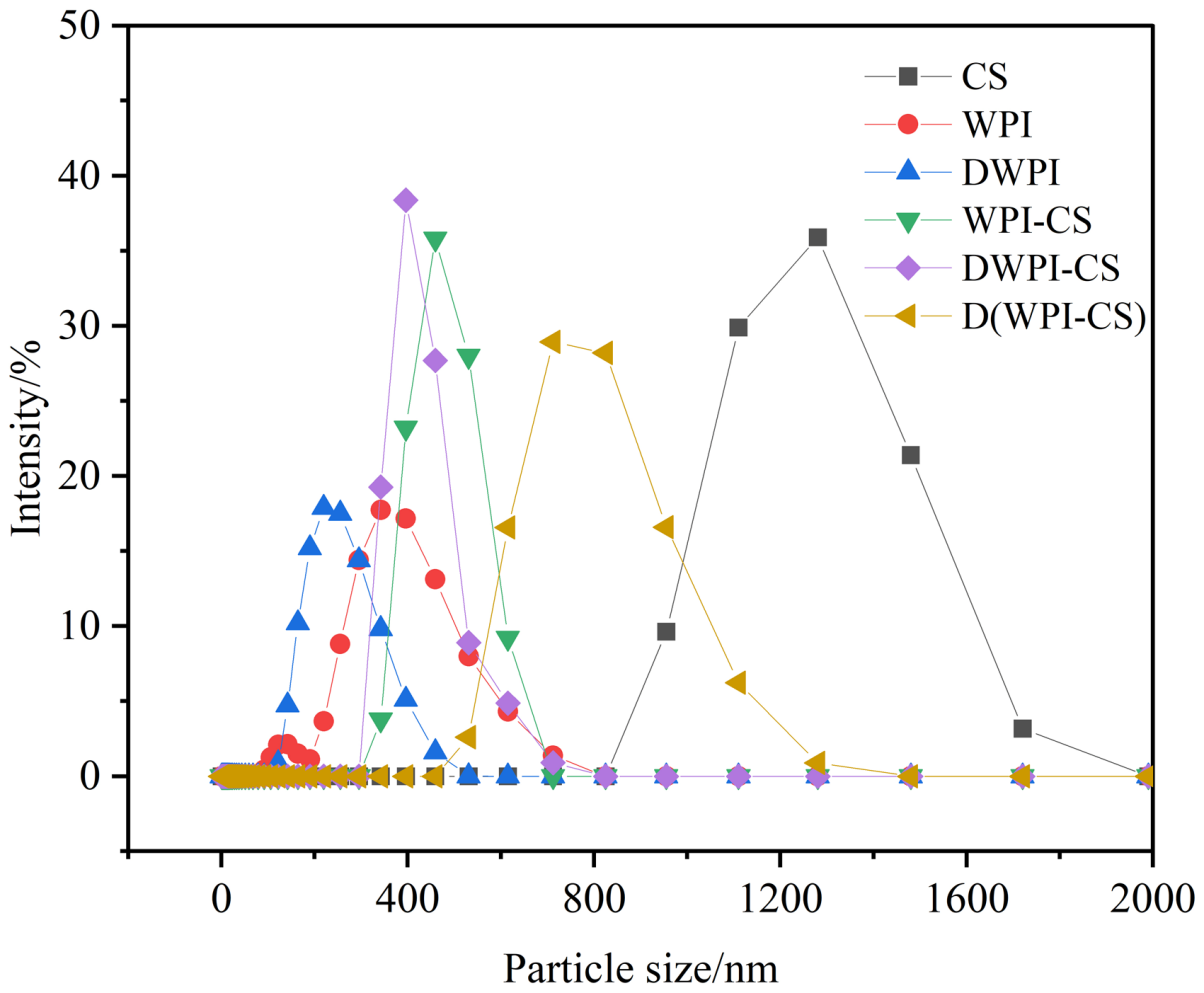
Particle size distribution of CS, WPI, DWPI, and their composite particles.

The particle size distribution diagrams of CS, WPI, DWPI, and the three types of WPI and CS composite particles are presented in Figure 2, and the average particle size and ζ potential values are listed in Table 1. Significant differences were observed in the particle sizes between sample types. The mean particle size of CS was 1228.00 ± 83.14 nm, while that of WPI was only 250.83 ± 8.37 nm, close to the mean particle size of WPI (253.8 nm) measured by Zhou et al. (28). The mean particle size of WPI decreased to 185.17 ± 6.41 nm after thermal denaturation. The same trend was also reported by Zembyla et al. (39); they revealed that the soluble component of WPI had a hydrodynamic diameter of 218 nm, which significantly decreased to 175 nm after heat treatment at 90°C (*p* < 0.05). The average size of the WPI-CS composite particles was 762.00 ± 7.81 nm, slightly lower than the average particle size of DWPI-CS. The average particle size of D(WPI-CS) was significantly larger than those of WPI-CS and DWPI-CS.

**Table 1:** Particle sizes, PDI, and ζ potentials of CS, WPI, DWPI, and their composite particles.

Particles	Size/nm	PDI	ζ potential/mV
CS	1228.00 ± 83.14a	1.00 ± 0.00a	21.10 ± 1.98a
WPI	250.83 ± 8.37d	0.19 ± 0.02d	−26.55 ± 2.67f
DWPI	185.17 ± 6.41e	0.25 ± 0.04d	−0.26 ± 0.24b
WPI-CS	762.00 ± 7.81c	0.45 ± 0.10c	−1.35 ± 0.06c
DWPI-CS	878.53 ± 7.85b	0.55 ± 0.43c	−6.45 ± 0.80e
D(WPI-CS)	1320.50 ± 0.71a	0.60 ± 0.02b	−1.75 ± 0.19d

Different letters in the same column indicate significant differences in the related indicators between different particles (*p* < 0.05).

CS is the only natural cationic polysaccharide tested in our study; therefore, its ζ potential had a positive value. WPI is a negatively charged protein with a ζ potential of −26.55 ± 2.67 mV. Conversely, the charge of DWPI changed to −0.26 ± 0.24 mV after thermal denaturation. The ζ potential of WPI-CS was −1.35 ± 0.06 mV due to the formation of electrostatic bonds between the positively charged CS and negatively charged WPI, resulting in a decrease in the ζ potential. The ζ potential values of D(WPI-CS) and WPI-CS were comparable and were slightly lower than that of DWPI-CS (−6.45 ± 0.80 mV).

#### 3.1.2 SEM results

**Figure 3:**
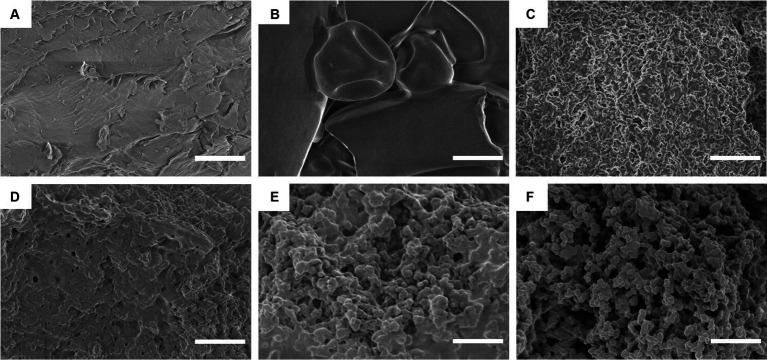
SEM images of CS, WPI, DWPI, and their composite particles. Images **A–F** refer to CS, WPI, DWPI, WPI-CS, DWPI-CS, and D(WPI-CS), respectively. The scale bars correspond to 5 μm.

SEM can be used to directly observe the microstructure and morphology of composite particles and is an advanced method for characterizing sample structures (41). In Figure 3, the SEM images of the different particles show significantly different morphological characteristics. CS has a flat and dense morphology, while WPI shows a typical spherical structure. However, DWPI exhibits a significantly different morphology from WPI, with a relatively uniform and dense structure. In contrast, the SEM images of the WPI and CS composites exhibited different morphological structures compared to those of CS and WPI. The morphologies of the DWPI-CS and D(WPI-CS) particle composites are similar, with porous network structures and uniform pores (39). The special structure of the WPI composite enhances its spatial stability, enabling its combination with large volumes of water and thereby improving its ability to stabilize the emulsion.

#### 3.1.3 AFM results

**Figure 4:**
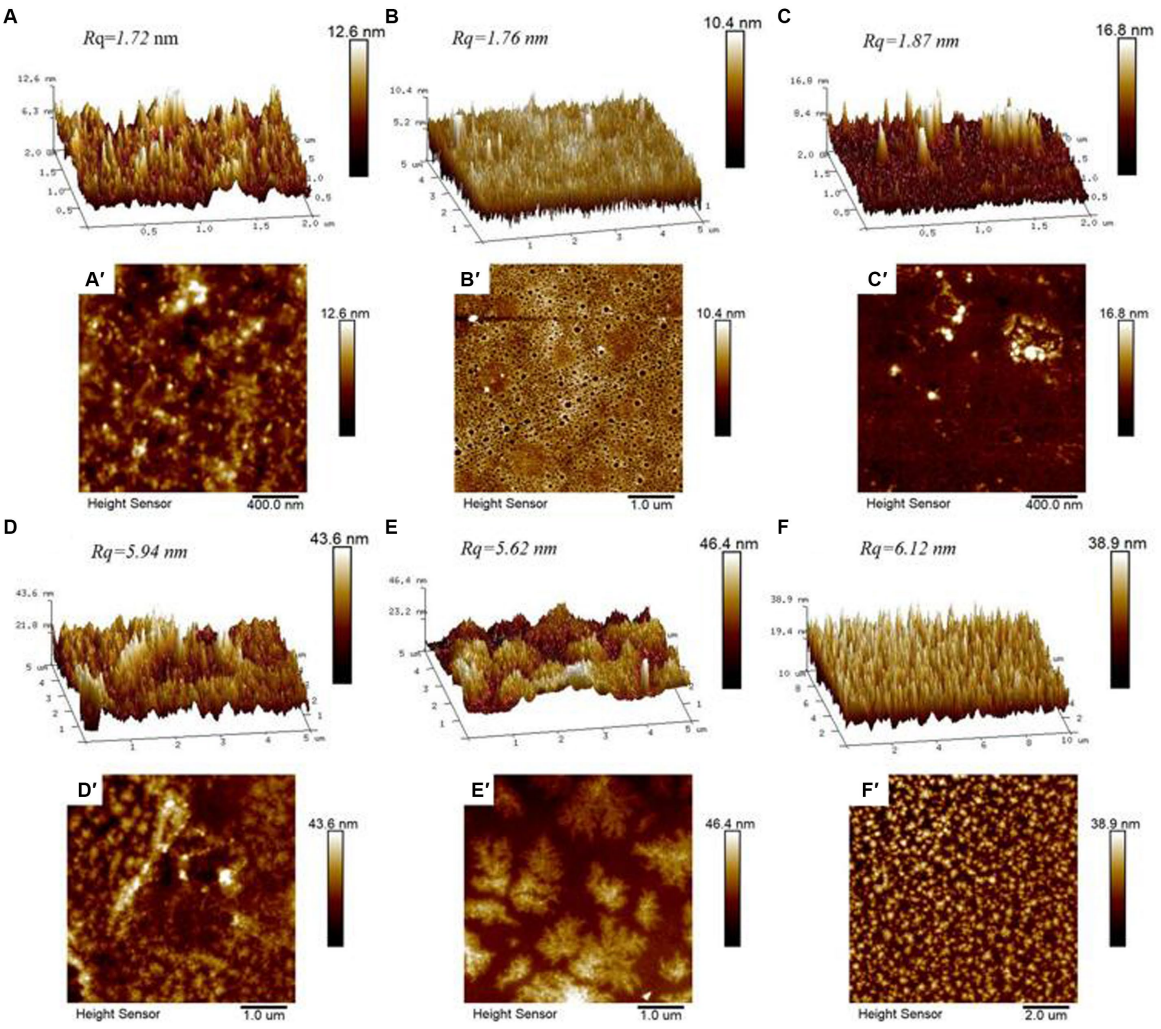
AFM three-dimensional image (above) and height diagram (below) of CS **(A,A’)**, WPI **(B,B’)**, DWPI **(C,C’)**, WPI-CS **(D,D’)**, DWPI-CS **(E,E’)**, and D (WPI-CS) **(F,F’)**.

**Figure 5:**
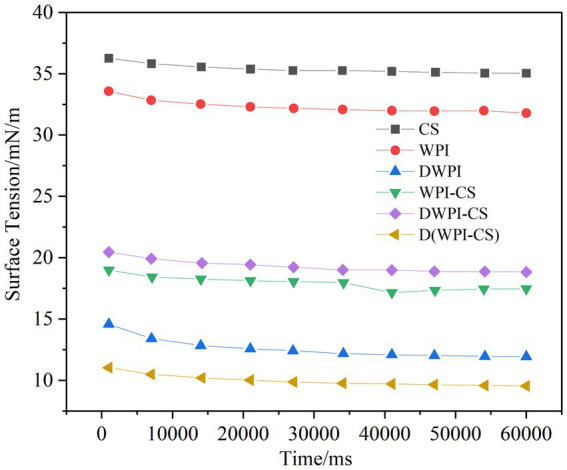
Interfacial tension of CS, WPI, DWPI, and their composite particles at the oil–water interface.

AFM was used to characterize the morphology and thickness of the particles, as well as the adsorption morphologies of CS, WPI, DWPI, and their composite particles (Figure 4). The images show that the surfaces of the samples exhibit a fluctuating morphology. Relative to the AFM images of the CS and WPI composite particles, the “peak” shapes of CS, WPI, and DWPI appear “sharper” and “slimmer,” with lower surface roughness values of 1.72, 1.76, and 1.87 nm, respectively. The morphologies of the WPI and CS composites show significant changes compared to those of the individual emulsifiers, forming relatively large aggregates with surface roughness values of 5.94, 5.62, and 6.12 nm, respectively. The contour size and particle height increased significantly, as shown in Figure 5, indicating that the desorption energy of the particles adsorbed on the O/W interface increased. Based on the Pickering stability mechanism (42), the stability of the emulsions stabilized by the WPI and CS composite particles increased compared to that of those stabilized by CS or WPI alone. In addition, the distribution of the “peak” shape of the D(WPI-CS) composite was much more uniform based on the observed morphological characteristics.

#### 3.1.4 Interfacial tension results

A low interfacial tension is conducive to the stability of the emulsion, with the adsorption of emulsifiers at the oil–water interface reducing the interfacial tension (43). The typical dynamic interfacial tension curve of a protein emulsifier comprises two phases (44): in the initial stage, the phase has a steep slope as the protein emulsifier moves toward the oil–water interface and is adsorbed on it. At this stage, the proteins undergo conformational changes and rearrangements. In the second stage, the phase exhibits an almost horizontal slope corresponding to the stability of the protein at the interface. The interfacial tension between the aqueous phase of the different emulsifiers and the oil phase of the MCT used in this study is presented in Figure 5, revealing a much higher interfacial tension of the oil–water phase of the single-component CS and WPI compared to the other emulsifiers. The dynamic interfacial tension of the WPI and WPI/CS composite particles is representative of the typical changes in protein emulsifiers. Compared to WPI, the interfacial tension of the thermally modified WPI is significantly reduced owing to the exposure of more hydrophobic amino acids in WPI after heat treatment, making it easier for the proteins to arrange themselves on the surface of the oil droplets, resulting in a decrease in the interfacial tension between oil and water.

These results are consistent with the findings of Wang et al. (45) on the influence of heat treatment on the interfacial tension between WPI liposomes and soybean oil. The oil–water interfacial tensions of WPI-CS, DWPI-CS, and D(WPI-CS) decreased significantly compared to those of CS and WPI. D(WPI-CS) exhibited the lowest interfacial tension among the three composite particles, followed by WPI-CS and DWPI-CS. These results indicate that the interaction between WPI and CS after complexation can change the adsorption capacity of WPI at the oil–water interface. This observation may be due to the increased exposure of hydrophobic groups in WPI after complexation with CS, facilitating inward stretching into the oil phase. The composite particles were arranged at the oil–water interface, thereby decreasing the interfacial tension (45).

#### 3.1.5 XRD results

**Figure 6:**
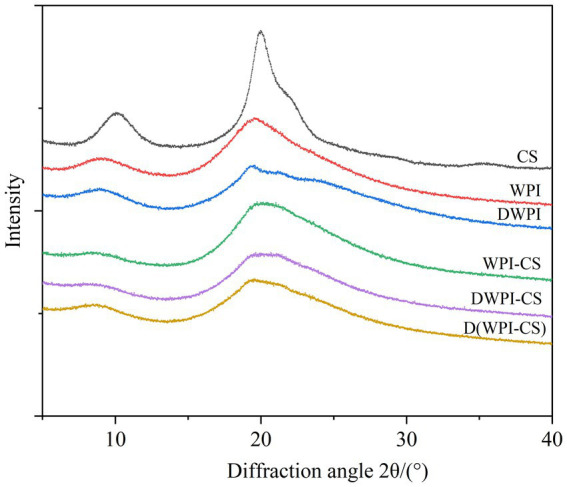
XRD patterns of CS, WPI, DWPI, and their composite particles.

The structural characteristics of CS, WPI, DWPI, and their composite particles were measured using XRD with diffraction angles ranging between 5–40°. The results (Figure 6) show clear peaks for CS at 2*θ* values of 10.04° and 19.98°, indicating that CS is highly crystalline, which is consistent with the results of previous studies (46). WPI shows a relatively wide peak at a 2*θ* value of 19.54° and a small peak at 9.02°, which are representative of the protein’s α-helix and β-sheet structure. The characteristic peak of DWPI undergoes slight changes; it shifts to 19.58° and the peak shape narrows. The corresponding 2*θ* value for the small peak shifts to 8.92°, indicating that the protein’s secondary structure is changed by thermal denaturation, which alters its crystal form. In the XRD spectra of the three composite particles, the two characteristic peaks of CS are not observed, indicating that CS is enveloped in the composite particles in an amorphous manner. The characteristic peak of WPI-CS undergoes a slight shift compared to that of WPI, migrating to 19.74°, and the small absorption peak around 9° is significantly weakened. The characteristic peak of DWPI-CS shifts significantly compared to that of DWPI, with the 2*θ* value reaching 20.68° with a wide peak deformation. The peak shape of D(WPI-CS) is similar to that of DWPI and the characteristic peak position is ~19.52°.

#### 3.1.6 FTIR spectroscopy analysis

**Figure 7:**
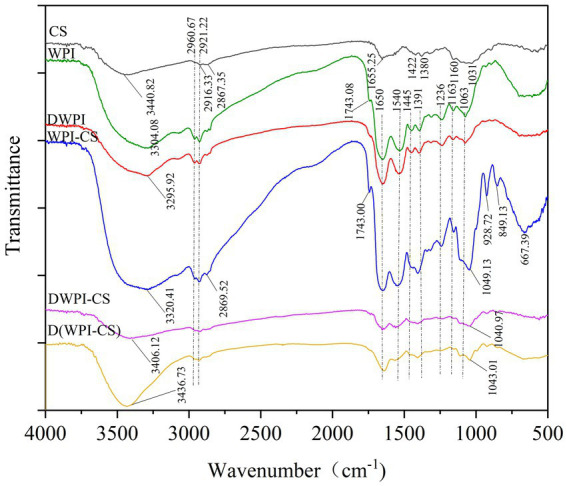
FTIR spectra of CS, WPI, DWPI, and their composite particles.

FTIR analysis revealed molecular interactions between WPI and CS. Figure 7 presents the FTIR spectra of CS, WPI, DWPI, and their composite particles. The infrared spectrum of CS shows a characteristic peak at approximately 3,440 cm^−1^ indicative of a robust amino N–H bond, with a C–H bond stretching vibration at 2916.33 cm^−1^ and amide III (–NH^3+^)-related bands at 1,650 cm^−1^; these contributed to the vibration of –OH and –CH groups at 1,422 cm^−1^, symmetric stretching of C–O–C at 1,160 cm^−1^, and stretching vibration of C at 1,063 cm^−1^. Such findings are consistent with previous reports (47). In the infrared spectrum of WPI, the broad peak near 3,304 cm^−1^ is attributed to the stretching vibration of hydroxy (OH) or amino (N–H) bonds; the peaks at 2960 and 2,922 cm^−1^ are, respectively, attributed to the antisymmetric stretching vibration and symmetric stretching vibration of C–H bonds in methyl or methylene groups; the 1743.08 cm^−1^ peak is assigned to the stretching vibration of carbonyl (C=O) bonds. Following thermal denaturation, the hydroxyl peak of WPI shifts from 3304.08 to 3295.92 cm^−1^, which may be due to the influence of thermal denaturation on the hydrogen bonding of the protein molecules. The intensity of the C=O peak at 1,745 cm^−1^ is significantly reduced, indicating that thermal denaturation disrupts the C=O bond structure in WPI. Furthermore, heat treatment led to interactions between the whey protein molecules and partial loss of their intramolecular structure. A similar change in the secondary structure of whey protein was observed in a previous study when a BiPro^®^ WPI dispersion (10% w/v) was heated from 70 to 90°C in water at pH 7 (48); it was speculated that the intermolecular interactions established during heating were due to the formation of hydrophobic bonds on the surface of the dimer (I strand; His146 Ser150) (49).

The infrared spectrum of the WPI-CS composite particles showed a significant enhancement in the C=O peak intensity at 1,745 cm^−1^ compared to that of WPI and DWPI, possibly due to the tensile vibration between the amino group (–NH^3+^) of CS and the carbonyl group (C=O) of the carboxyl group (–COO–) of WPI, indicating an electrostatic interaction between the amino group of CS and the carboxyl group of WPI (46). Compared to the spectra of WPI and CS, the spectrum of the WPI-CS composite particles showed a wide band at approximately 3,000–3,600 cm^−1^. These results indicate that hydrogen bonding is involved in the interaction between WPI and CS and that the hydrogen bonding effect is enhanced, which is consistent with previous studies (42). Furthermore, the WPI-CS composite particles exhibited new peaks at 928.72, 849.13, and 667.39 cm^−1^. The new peak at 849.13 cm^−1^ may be caused by the C–H angular vibration of the CS glycosidic bond, while that at 667.39 cm^−1^ may be caused by the bending vibration outside the O–H of CS. Compared with WPI-CS, the C=O peak intensities of DWPI-CS and D(WPI-CS) were significantly lowered near 1,743 cm^−1^, and were lower than those of WPI and DWPI, respectively. Furthermore, the peak intensities of DWPI-CS and D(WPI-CS) were significantly lowered at 1,540, 1,445, and 1,236 cm^−1^, indicating that compared with the WPI-CS composite particles, the interactions between CS and the denatured WPI complex were weakened. Such a result indicates that the thermal denaturation of WPI leads to changes in the spatial structure of the protein, affecting the molecular interaction between WPI and CS. In addition, the C–O bonds of the three composite particles of WPI and CS moved to approximately 1,049 and 1,063 cm^−1^, possibly due to the C–OH stretching vibration of the hydroxyl groups of CS. The peak intensity of D(WPI-CS) near 3,400 cm^−1^ was significantly higher than that of DWPI-CS and the peak migrated to 3436.73 cm^−1^.

### 3.2 Evaluation of the emulsions stabilized by WPI and CS composite particles

#### 3.2.1 Appearance, optical image, and droplet size

**Figure 8:**
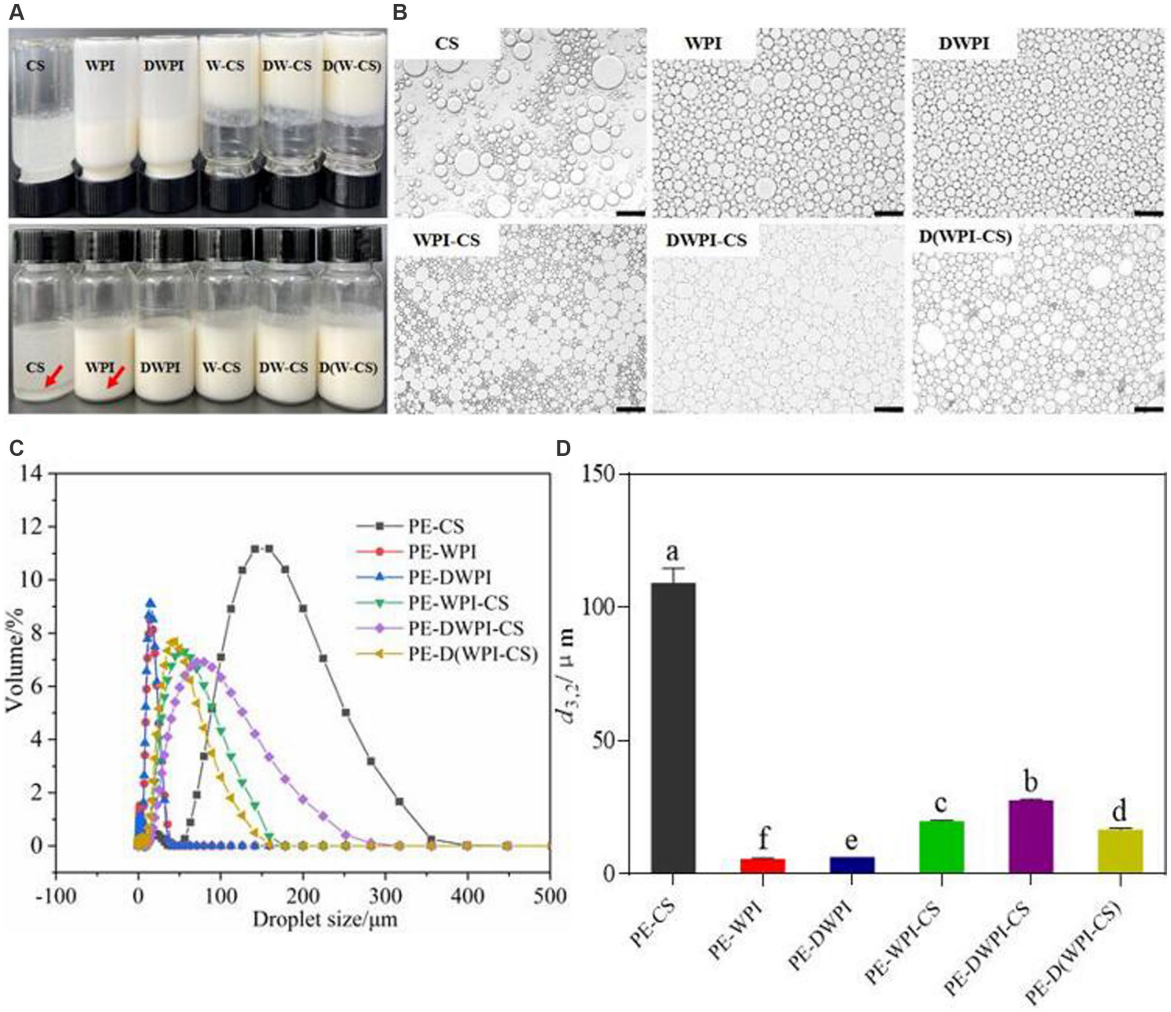
Appearance **(A)**, optical images **(B)**, droplet size distribution diagram **(C)**, and average droplet size *d*_3,2_ **(D)** of emulsions stabilized by CS, WPI, DWPI, and their composite particles. Scale bars correspond to 100 μm. The letters a–f indicate statistically significant differences between these samples (*p* < 0.05). PE means Pickering emulsion.

The sensory observations and microstructures of the emulsion samples stabilized with different particles are shown in Figures 8A,B. The emulsion samples prepared by CS show notable stratification; an oil–water separation phenomenon occurs, indicating that a stable emulsion cannot be prepared using CS alone. Large, spherical oil droplets can be observed in the microstructure, with large distances between the oil droplets. The emulsion prepared with WPI and DWPI exhibits a “backflow” phenomenon after the glass bottle is inverted, while the three groups of emulsions stabilized with WPI and CS composite particles do not. Observation of the microstructure shows that the emulsion droplets stabilized by WPI or DWPI alone are spherical. In contrast, the microstructure of the emulsions stabilized by the WPI and CS composite particles is polygonal, showing the difference in particles on the oil–water interface between the composite emulsifiers and WPI (when used alone). The droplet size distribution diagrams and average droplet size (*d*_3,2_) values of the emulsions stabilized with CS, WPI, DWPI, and their composite particles are shown in Figures 8C,D. The emulsion stabilized with CS exhibited the largest droplets (106.41 ± 3.86 μm). These results are consistent with the optical microscopy results described in section 3.8. The particle size values of the emulsions stabilized with WPI and DWPI are similar, at 6.01 ± 0.01 μm and 6.59 ± 0.00 μm, respectively. The average droplet size of the emulsions stabilized with the three types of WPI and CS composite particles are 20.00 ± 0.15 μm, 27.80 ± 0.35 μm, and 16.77 ± 0.51 μm, respectively.

#### 3.2.2 Rheological properties

**Figure 9:**
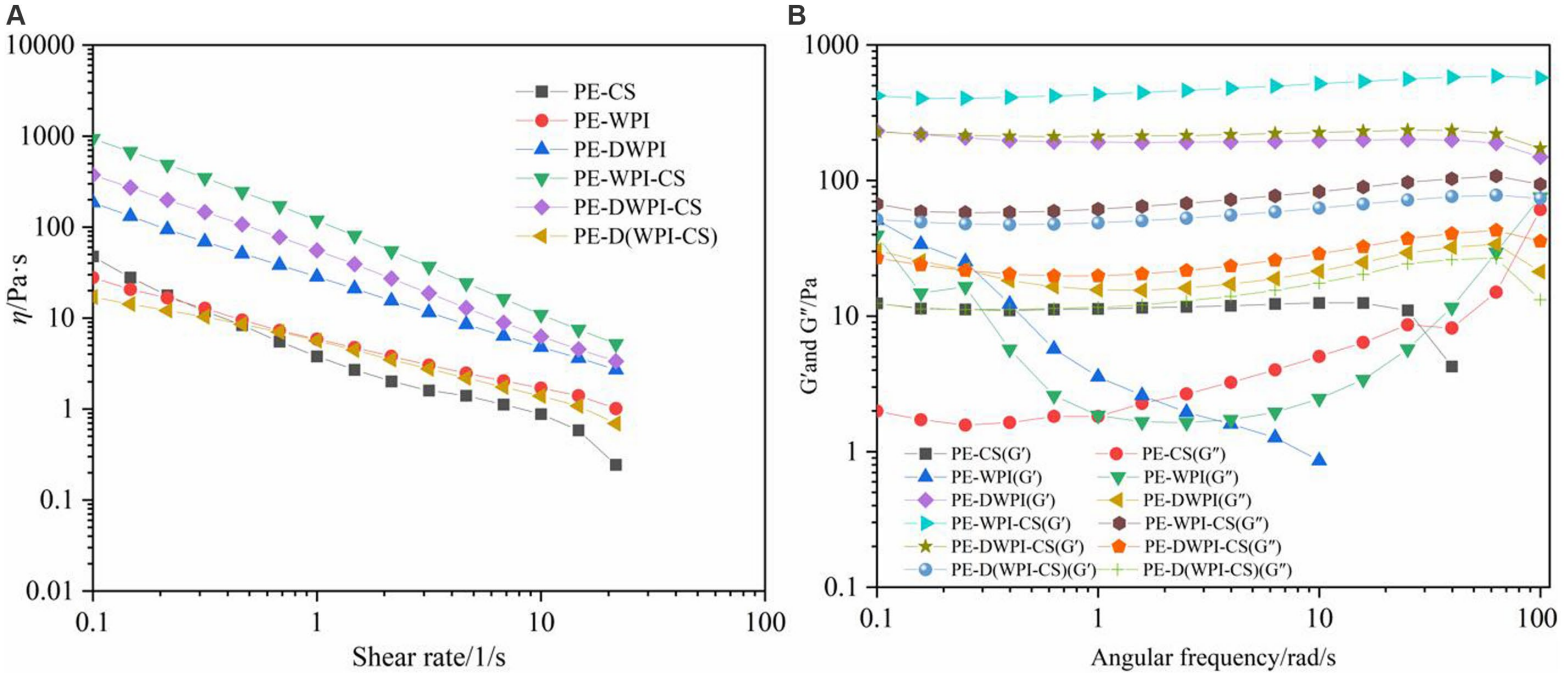
Apparent viscosity **(A)**, storage modulus (*G′*), and loss modulus (*G′*′) **(B)** of emulsions stabilized by CS, WPI, DWPI, and their composite particles.

Rheological properties have an important influence on the stability of emulsions, and can explain the microstructure of emulsions (27). The rheological properties of conventional emulsions (such as emulsions stabilized with the non-ionic surfactant Tween 80/Span 80) are usually determined by the continuous phase; however, in Pickering emulsions, the distance between the oil droplets is shorter, and the stabilizer particles have a more significant impact (27). The rheological properties of the emulsions stabilized with CS, WPI, DWPI, and their composites are shown in Figure 9. Figure 9A shows the apparent viscosity (*η*), which decreases for all emulsions with an increase in the shear rate; this is a characteristic of pseudoplastic emulsions in non-Newtonian fluids (9, 10). As shown in Figure 9B, except for the emulsions with CS or WPI, the storage moduli (*G*′) of the other emulsion samples are greater than the corresponding energy dissipation moduli (*G*″) over the entire frequency range, indicating that the emulsions are predominantly elastic and exhibit a 3D network structure (32). In contrast, in the rheological curves of emulsions stabilized with CS or WPI, *G*″ is greater than *G*′, indicating that these two emulsion samples have unstable characteristics, consistent with the sensory evaluation and microstructural observation results. In addition, the apparent viscosity, *G*′, and *G*″ of the emulsion stabilized with WPI-CS are higher than those of the other emulsion samples, indicating its higher stability. This conclusion is consistent with the FTIR results, indicating that the hydrogen bond formed between WPI and CS is stronger compared to those of the other composite emulsifying agents; furthermore, its ability to stabilize an emulsion is enhanced.

### 3.3 Thermal stability and biological accessibility of the curcumin emulsion

#### 3.3.1 Thermal stability results

**Figure 10:**
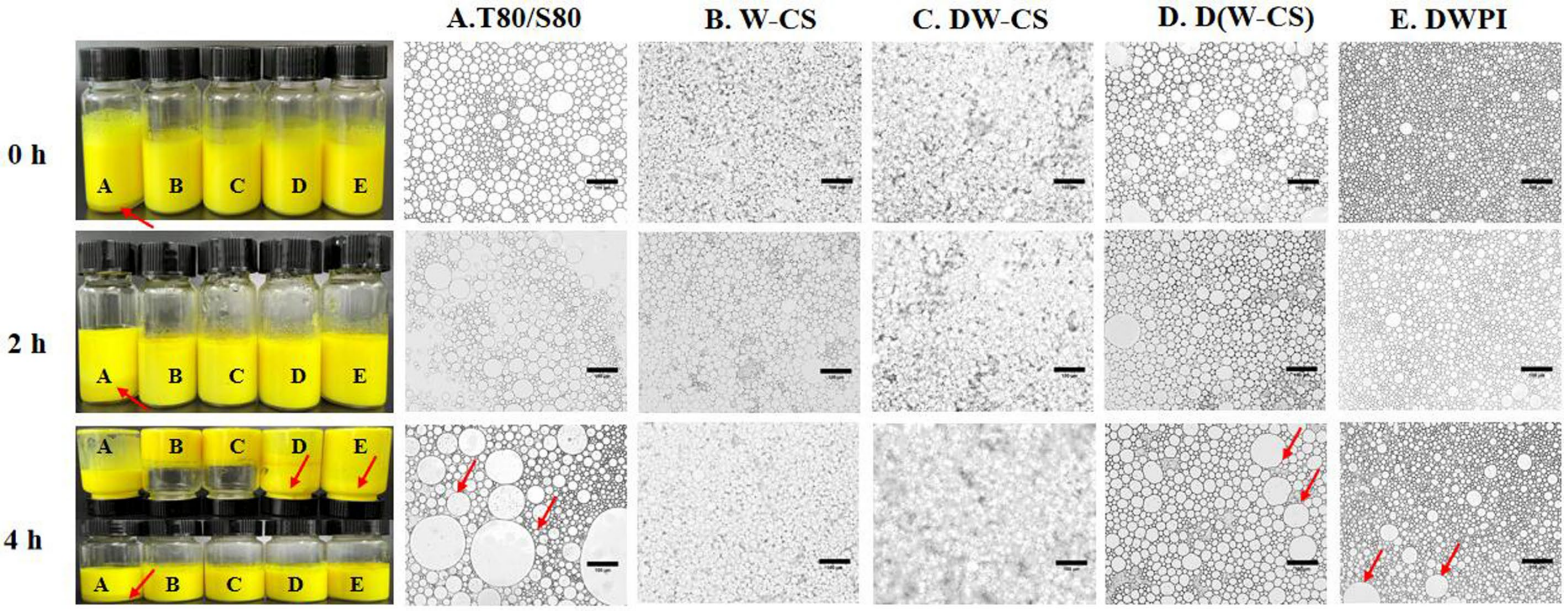
Visual appearance (left) and optical image (right) of curcumin in emulsions at 80°C for 4 h. The scale bars of the optical micrographs correspond to 100 μm.

Samples of Pickering emulsion-encapsuled curcumin stabilized with WPI-CS were placed in a water bath at 80°C for 4 h. Their appearance and microstructures are shown in Figure 10. It is obvious that at the initial stage (0 h), the control group samples based on Tween 80 and Span 80 showed obvious stratification, while the Pickering emulsion samples based on WPI-AXs were uniform and free of stratification, exhibiting curcumin’s unique yellow color. After being placed at 80°C for 4 h, the sample bottle was inverted. The Pickering emulsions with DWPI-CS and WPI-CS did not flow down, whereas the other groups exhibited “backflow” phenomena. The microstructure diagram shows a significant increase in the oil droplet size of samples in the DWPI and D(WPI-CS) groups after being placed at 80°C for 4 h, while the microstructures of the Pickering emulsions in the other groups did not change significantly. This finding was consistent with the observed changes in sample appearance, indicating that the emulsions stabilized with WPI-CS and DWPI-CS have relatively better thermal stability than the other Pickering emulsion samples, which is consistent with their rheological properties.

#### 3.3.2 Biological accessibility of curcumin in emulsion

**Figure 11:**
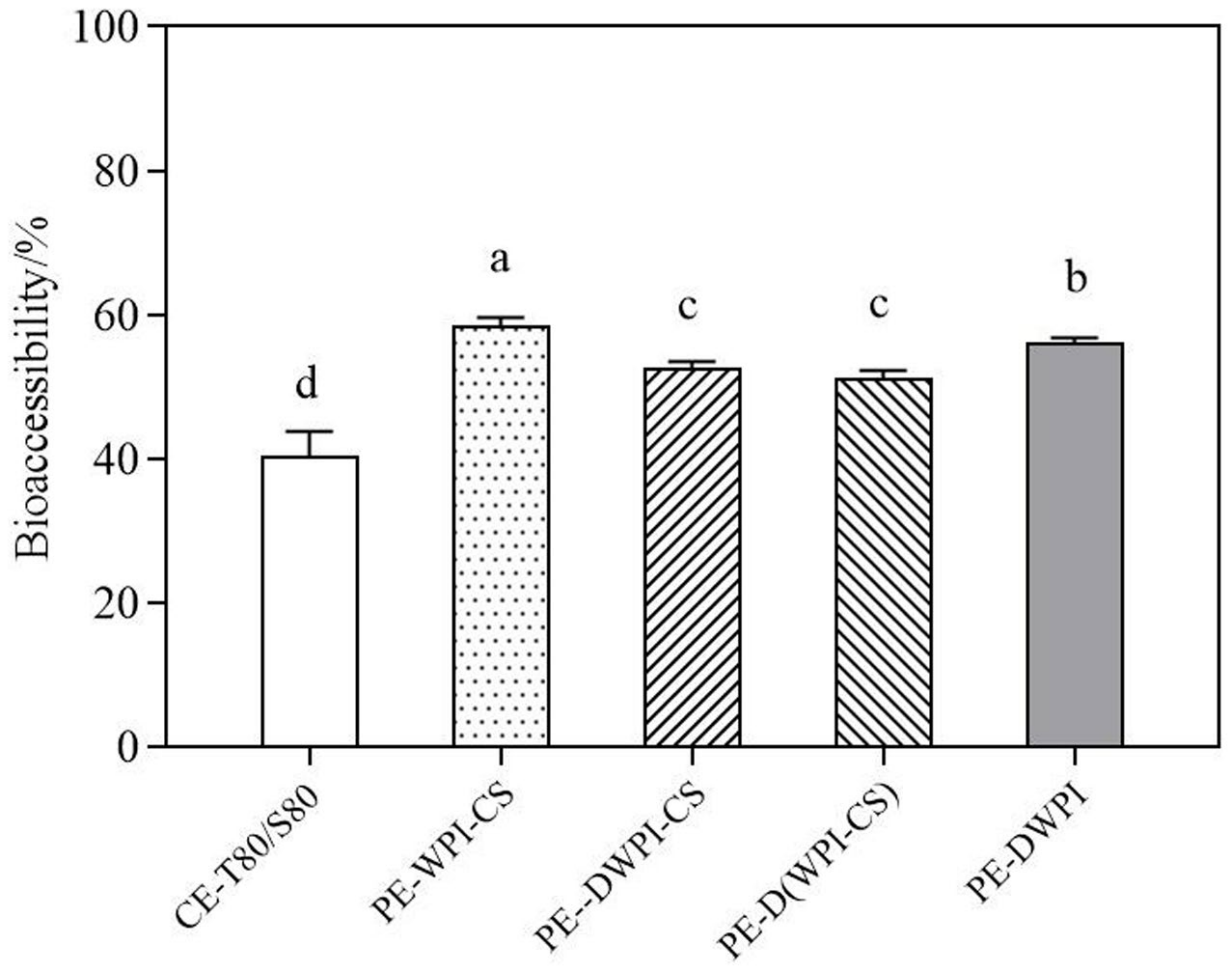
Comparison of the bioaccessibility of curcumin between the emulsion samples. The letters a–d indicate the significant differences between the different samples (*p* < 0.05).

Figure 11 presents the bioaccessibility of curcumin in emulsions with different particles after simulated GIT digestion. The bioaccessibility of curcumin in Pickering emulsions was significantly higher than that of the conventional emulsion stabilized with Tween 80 and Span 80. As aforementioned, the mixed micelles formed by lipids and hydrophobic compounds are essential for the absorption of many hydrophobic compounds (50). Compared with the conventional emulsion, the Pickering emulsion improved the *in vitro* bioaccessibility of curcumin. In addition, the interactions between peptides generated by protein hydrolysis may also contribute to micellization, which is conducive to the dissolution of curcumin. In Pickering emulsions, the bioaccessibility of curcumin in PE-WPI-CS was the highest, reaching 61.18 ± 0.16%, followed by PE-DWPI, which was significantly higher than that of PE-DWPI-CS and PE-D(WPI-CS). Meanwhile, in the *in vitro* experiments, no significant difference was observed in the bioaccessibility of curcumin between DWPI-CS and D(WPI-CS) (*p* ˃ 0.05), indicating that the composite particles formed by heat denatured WPI and CS, and the composite particles formed by heat denaturation after mixing with WPI and CS, exhibit no significant difference in their *in vitro* digestion effect.

## 4 Conclusion

Three types of composite particles were prepared to study the effect of heat on the composition of WPI and polysaccharides and reveal the stabilizing mechanism of their emulsions. The particles were characterized via analyses of their particle size, ζ potential, SEM, AFM, XRD, FTIR, and interfacial tension measurements. Pickering emulsions were prepared with the aforementioned composite particles and evaluated, revealing that CS was compatible with WPI or DWPI; composite particles prepared using WPI and DWPI exhibited a stronger emulsifying ability than WPI or CS alone. The average droplet size of the emulsions stabilized with the three types of WPI and CS composite particles were 20.00 ± 0.15 μm, 27.80 ± 0.35 μm, and 16.77 ± 0.51 μm, respectively. The stability mechanism was attributed to the molecular interactions between WPI and CS, increasing the desorption energy of the composite particles adsorbed on the O/W interface and a decrease in the oil–water interfacial tensions of the composite particles. Thermal stability experiments showed that the curcumin emulsions stabilized with WPI-CS and DWPI-CS have relatively better thermal stability than the other samples; such a finding is consistent with the results obtained from rheological tests. *In vitro* bioaccessibility experiments showed that the bioaccessibility of the curcumin emulsion stabilized with WPI-CS was 61.18 ± 0.16%, significantly higher than that of the emulsions prepared with the other two composite particles (*p* < 0.05). This study provides insights into the design of customized WPI and CS composite-based Pickering emulsions for the food and nutrition industries. Future work should include application of the composite particle-based Pickering emulsions in the delivery of active ingredients and evaluation of their encapsulation efficiency, drug-loading capacity, storage stability, digestion rate, and bioavailability.

## Data availability statement

The raw data supporting the conclusions of this article will be made available by the authors, without undue reservation.

## Author contributions

YP: Data curation, Formal analysis, Investigation, Writing – original draft, Writing – review & editing. YL: Data curation, Formal analysis, Investigation, Writing – original draft, Writing – review & editing. DX: Data curation, Formal analysis, Investigation, Writing – original draft, Writing – review & editing. YN: Formal analysis, Writing – original draft, Writing – review & editing. QW: Formal analysis, Writing – original draft, Writing – review & editing. SC: Formal analysis, Writing – original draft, Writing – review & editing. RW: Formal analysis, Writing – original draft, Writing – review & editing. RG: Conceptualization, Investigation, Data curation, Formal analysis, Writing – original draft, Writing – review & editing.
